# Unveiling a Rare Case of a Giant Aggressive Angiomyxoma of the Perineum: Diagnostic Insights From T2-Weighted MRI

**DOI:** 10.7759/cureus.78971

**Published:** 2025-02-13

**Authors:** Tarang Patel, Virendrakumar Meena, Parth R Goswami, Gyanendra Singh

**Affiliations:** 1 Pathology, All India Institute of Medical Sciences (AIIMS) Rajkot, Rajkot, IND; 2 Radiology, Geetanjali Medical College & Hospital, Udaipur, IND

**Keywords:** aggressive angiomyxoma, angiomyofibroblastoma, hypocellular tumor, magnetic resonance imaging, myxoid background, perineal mass

## Abstract

Aggressive angiomyxoma (AA) is a rare benign soft tissue tumor seen in females of the reproductive age group and involving the lower pelvis and perineal soft tissue. It is characterized by the presence of both myxoid and vascular components. We present a case of a 36-year-old female who presented with right buttock swelling. Magnetic resonance imaging (MRI) revealed a large perineal swelling suspected to be a giant AA, which was demonstrating hypocellular stroma with a myxoid background in biopsy. AA should be suspected in a female patient presented with large perineal swelling. MRI should be the investigation of choice for radiological diagnosis of this rare entity to be confirmed by a pathology report.

## Introduction

Aggressive angiomyxoma (AA) is a benign soft tissue tumor with myxoid and vascular components arising mostly in the lower pelvis and perineum in women of reproductive age (third to fifth decades of life) [1,2]. Several recent studies indicate that AA may be linked to changes in the chromosome structure in the 12q13-15 region, as well as abnormal expression of the HMGIC gene (a gene involved in DNA architecture) caused by a specific chromosomal translocation known as t(8;12) [3]. Microscopically, a tumor is composed of scattered spindle cells and abundant medium-sized vessels, embedded in the myxoid matrix [4].

AA is called aggressive because of the extensive local extension and post-operative local recurrence. Two factors are responsible for the high rate of local recurrence; first, the tumor is generally not diagnosed before initial surgery and the extent of the tumor is also not perceived frequently. Second, the tumor is located around the urethra, vagina, anal sphincter, and rectum and extends above and below the pelvic diaphragm. The difficulty in achieving total resection from pelvic or abdominal approaches alone is attributed to these factors, leading to either inadequate resection or residual tumor [4,5].

Imaging techniques, with a specific emphasis on magnetic resonance imaging (MRI), are crucial in the clinical management of AA. The results obtained from MRI are not only indicative of the disease but also aid in quantifying its growth extent. In addition to facilitating the surveillance of response to potential preoperative interventions, such as hormone therapy, MRI remains indispensable during post-treatment follow-up with the patient [6].

AA is frequently misdiagnosed at first as gynecological cancer or a groin hernia, resulting in needless surgical treatments. The primary therapeutic approach for AA remains the complete surgical removal of the tumor with clear margins [7].

## Case presentation

**Figure 1:**
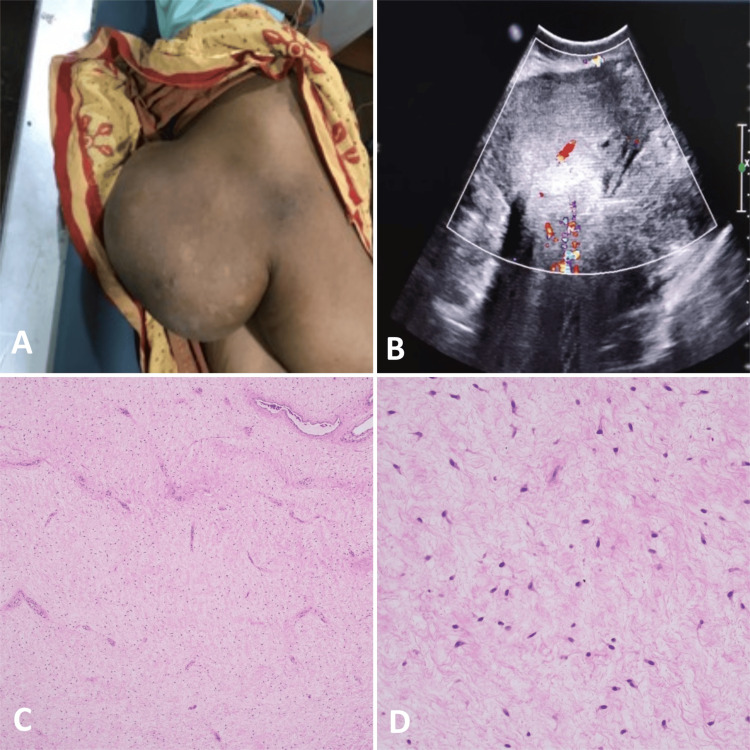
Clinical, ultrasound sonography (USG), and histopathological findings. (A) Large lump in the gluteal and perineal region.  (B) Large heteroechoic mass lesion in the gluteal and perineal region with raised vascularity on color Doppler. (C) Low-power view and (D) high-power view showing a pauci-cellular tumor having bland stellate cells with myxoid stroma. (HE, 10x & 40x)

A 36-year-old female presented to the oncology department of a tertiary care center in Udaipur, India, with the chief complaint of swelling over the right buttock for the past year and constipation for the past three months. There was no history of any per-rectal or per-vaginal bleeding. There was no history of any hematuria or any other significant relevant history. Complete blood count, liver function test, kidney function, and viral markers revealed no significant abnormality. On examination, there was a soft consistency lump in the gluteal and perineal region (Figure 1A). On per-rectal examination, it was a soft mass pushing the rectum towards the left side with no obvious rectal infiltration. Ultrasound was done for the large mass lesion, which shows a heteroechoic echotexture with raised vascularity (Figure 1B).

**Figure 2:**
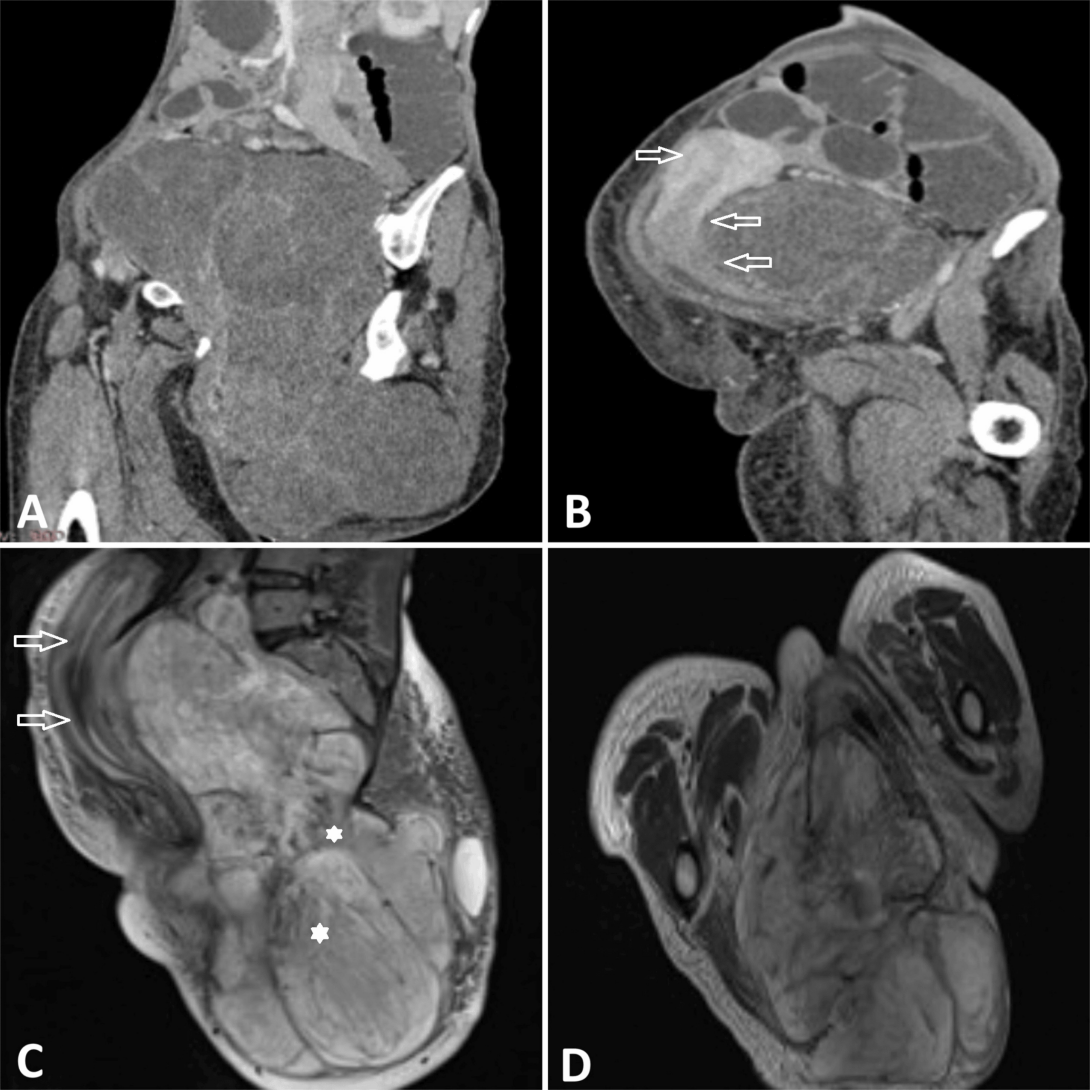
Imaging findings (A & B) CT images show contrast-enhanced sagittal reformated images showing a hypodense mass lesion in the pelvic cavity, perineum, and gluteal region with few foci of septal enhancement within the tumor. The mass lesion is causing anterior displacement of the uterus, cervix, upper vagina, and urinary bladder without any infiltration. (Uterus, cervix and upper vagina marked with arrows from upper to lower). (C & D) MRI images: Sagittal and axial T2 weighted spin echo images show a large high-signal-intensity mass lesion with lower-signal-intensity swirled strands (Indicated by asterisk) within the tumor. The uterus, cervix, upper vagina, and urinary bladder are displaced anteriorly (indicated by arrows).

The patient was further evaluated radiologically to determine the type of mass and its extent. On CT imaging, a large mass lesion was evident involving the pelvic cavity and perineum, measuring about 33 x 17 x 16 (CC x AP x T) (craniocaudal x anterior-posterior x transverse) cm with predominantly non-enhancing components with multiple enhancing vessels within the lesion (Figure 2A, 2B). Mass lesion was anteriorly displacing and compressing the uterus, cervix, upper vagina, and urinary bladder without any obvious infiltration. Furthermore, for better characterization of the lesion, an MRI was done, which showed a largely predominant T2 hyperintense mass lesion involving the perineum and pelvic cavity, suggestive of a myxoid content of mass (Figure 2C, 2D).

Finally, the patient was undertaken for a guided biopsy, and hematoxylin and eosin-stained sections showed a hypocellular tumor with a bland spindle to stellate cells in myxoid background, suggestive of AA. Nuclear atypia, hypercellularity, necrosis, or mitosis was absent (Figure 1C, 1D). The patient was scheduled to have a cystoscopy, followed by extensive excision of the tumor, while under general anesthesia. The surgical operation proceeded without any complications. On the third day post-operative, the patient was discharged with an uneventful recovery. Histopathological diagnosis was confirmed on surgical specimens with negative margin status microscopically. The patient demonstrated no recurrence/relapse on one-year follow-up imaging.

## Discussion

AAs are uncommon neoplasms that often manifest in adult females, with the highest occurrence observed between the ages of 30 and 50. The ratio of affected females to males is 6.6:1 [2]. Moreover, Steeper et al. [8] documented it for the first time in 1983. Approximately two years later, Begin et al. documented their findings on nine cases, which included the initial report of this condition in males [9]. The tumor commonly presents in the perineal and pelvic areas [10]. Rarely, they might be observed in male patients in associated areas, such as the scrotum and inguinal region [1].

Before surgery, radiographic exams such as ultrasound, computed tomography (CT), and MRI are used to assess the tumor's size and depth of infiltration into nearby organs and choose the appropriate surgical strategy based on the extent of the tumor [11]. They show on ultrasonography as isoechoic to hypoechoic mass with markedly increased vascularity [12]. A hypodense mass with well-defined boundaries and visible enhanced blood vessels can be observed on CT imaging [13]. Conversely, the tumor is usually very hyperintense on T2-weighted images. The presence of hyperintensity is likely caused by the abundance of water content and the loose myxoid matrix. The tumors appear with the same intensity as muscle in T1-weighted MRI scans. The mass exhibits distinct internal regions with a “swirled” linear pattern of low-intensity signal on both T1-weighted and T2-weighted imaging, which is caused by fibrovascular stroma. However, the distinct "swirled" look is not highly valued when observing contrast-enhanced CT scans [14].

When evaluating a patient with AA, the most frequent alternative diagnosis to consider is angiomyofibroblastoma. The two can be distinguished based on the size of the tumor. Angiomyofibroblastoma is characterized by modest size and affects only the superficial vulva and vagina. On the other hand, AAs typically include huge masses that impact the deep tissue planes. Myxomas and myxoid liposarcomas should also be regarded as significant alternative diagnoses. However, myxomas are situated inside the muscle tissue, whereas AA comes into contact with, but does not penetrate, the muscles of the pelvis and perineum. Myxoid liposarcomas primarily arise in the lower extremities, exhibit lacy or linear internal fat, and have homogenous enhancement. On the other hand, AAs do not have a considerable amount of internal fat and show heterogeneous enhancement [13,14].

A microscopic picture of the biopsy shows a hypocellular myxoid tumor having edematous stroma and many thin-walled blood vessels. Tumor cells have bland spindled to stellate appearance with minimal atypia. On Immunohistochemistry (IHC), tumor cells show positivity for vimentin, with variable reactivity for actin, desmin, and CD34 [15]. Wide local surgical excision is the treatment of choice for AA; sometimes, in the case of large tumors, hormonal therapies like raloxifene, tamoxifen, and gonadotropin analogs can be used to decrease the size of the tumor before surgery. Adjuvant treatment with gonadotropin-releasing hormone (GnRH) agonists has demonstrated encouraging outcomes in preventing recurrences. Chemotherapy and radiation have a limited role in the treatment of AAs owing to the low mitotic activity of the tumor [5,10,16].

Due to its rarity, AA is typically challenging to identify. However, a high index of suspicion must be kept in cases of pelvic masses in females of the fourth decade presenting with mass lesions in the pelvic region. Radiological imaging especially T2-weighted imaging and the location of the lesion are very important in making the diagnosis of the tumor. Such patients must be kept under long-term follow-up after surgical resection for local recurrence and relapse.

## Conclusions

We report this example to highlight the significance of MRI findings in diagnosing deep AAs. Therefore, healthcare professionals should employ MRI examination when faced with a situation involving a significant swelling in the perineal area of a female patient to detect this uncommon condition. The histological findings offer further validation of the radiological diagnosis.
